# A Serious Game for Young People With First Episode Psychosis (OnTrack>The Game): Qualitative Findings of a Randomized Controlled Trial

**DOI:** 10.2196/33526

**Published:** 2022-04-06

**Authors:** Samantha Jankowski, Kathleen Ferreira, Franco Mascayano, Effy Donovan, Reanne Rahim, Michael L Birnbaum, Sabrina Yum-Chan, Deborah Medoff, Bethany Marcogliese, Lijuan Fang, Terriann Nicholson, Lisa Dixon

**Affiliations:** 1 Department of Psychiatry New York State Psychiatric Institute New York, NY United States; 2 Department of Research and Evaluation C4 Innovations Needham, MA United States; 3 Department of Psychiatry The Zucker Hillside Hospital Glen Oaks, NY United States; 4 Department of Psychiatry University of Maryland School of Medicine Baltimore, MD United States; 5 Department of Psychiatry Columbia University Irving Medical Center New York, NY United States

**Keywords:** video gaming, internet, recovery, schizophrenia, psychosis, clinicians, mobile phone

## Abstract

**Background:**

Several studies have shown the benefits of coordinated specialty care (CSC) for individuals with first episode psychosis; however, pathways to care are marred by lack of knowledge, stigma, and difficulties with treatment engagement. Serious games or video interventions may provide a way to address these factors.

**Objective:**

This study focuses on qualitative results of a randomized controlled trial comparing OnTrack>The Game (OTG) with recovery videos (RVs) on engagement, stigma, empowerment, hope, recovery, and understanding of psychosis in clients receiving CSC. Clinicians are also interviewed regarding their perceptions of the interventions and suggestions for improvement.

**Methods:**

A total of 16 clients aged 16-30 years, with first episode psychosis attending a CSC program in New York State, and 9 clinicians participated in the qualitative interviews. Interviews were analyzed using the rapid identification of themes from audio recordings method.

**Results:**

For clients, themes included relatability of game content, an increased sense of hope and the possibility of recovery, decreased self-stigma and public stigma, increased understanding of the importance of social support, and increased empowerment in the OTG group. Clinicians had a preference for RV and provided suggestions for dissemination and implementation.

**Conclusions:**

Themes that may help inform future research in this area, particularly regarding dissemination and implementation of OTG and RV, emerged.

**Trial Registration:**

ClinicalTrials.gov NCT03390491; https://clinicaltrials.gov/ct2/show/NCT03390491

## Introduction

### Background

Schizophrenia can be a debilitating illness that affects approximately 20 million people worldwide [1]. Several studies have demonstrated the benefits of intensive early intervention programs, known in the United States as coordinated specialty care (CSC), for individuals with nonaffective first episode psychosis (FEP) [2-4]. However, treatment engagement can be challenging [5,6] and pathways to care are affected by stigma, symptom misattribution, and preference for self-management [7]. There is a need for tools that can help engage young people in treatment; reduce stigma; increase the understanding of psychosis, empowerment, and hope; and promote personal recovery.

A recent review has proposed the role of *serious gaming* as a way to promote engagement of individuals with psychosis, particularly if the game has a clear goal and involves service users in game development [8]. Serious games are those that have a clear educational purpose, can provide individuals with the opportunity to engage in decision-making for real-world situations in a safe environment, and help them envision positive future events and roles [8]. These games also have the potential to motivate individuals to engage in treatment and provide an avenue for disseminating mental health information in a manner that is nonthreatening or portrayed in a more casual and easily accessible way, rather than through formal clinician–patient contact [9]. A recent study by Ferchaud et al [10] has shown that self-identification with a video game avatar with psychosis reduced the desire for social distance from individuals with mental illness, thereby, providing the opportunity to reduce stigma. However, the impact of serious games on individuals with psychosis needs further examination, particularly owing to a dearth of randomized controlled trials (RCTs) in this area [11].

In a previous pilot study, we developed and tested a prototype of OnTrack>The Game (OTG), a computer-based role-playing game for individuals with FEP. We asked 20 individuals who are enrolled in OnTrackNY, a CSC program for individuals with FEP, to test the game in one 45-60–minute sitting [12]. The game included a customizable character, quests to practice real-world skills, information about FEP, and videos emphasizing stories of hope and recovery with individuals who have experienced FEP (ie, recovery videos [RVs]). The RVs have been previously shown to be effective in reducing public stigma in cross-sectional [13] and longitudinal [14] RCTs. Results from the OTG pilot study suggested a significant increase in positive attitudes toward recovery; however, we did not detect significant differences in hope, empowerment, and engagement. Moreover, qualitative results indicated that individuals found the community setting of OTG, educational components, and RVs to be the most helpful, and participants noted that playing OTG has the potential to translate to real-world decision-making.

In this study, we refine and augment OTG using clinician and client feedback (eg, Youth Advisory Board composed of individuals with FEP) and test it in an RCT comparing the short-term (2 months) and long-term (5 months) effects of OTG versus RVs on engagement, stigma, empowerment, hope, recovery, and understanding of psychosis. RVs are chosen as the comparison group owing to pilot study participants reporting RVs as the most valued game feature [12]. In this study, we want to determine whether the videos alone were producing an effect on our domains of interest or if the videos embedded in the game in addition to other game elements were creating an effect. RCT results will be presented separately. This paper focuses on the qualitative findings from OnTrackNY client and clinician interviews.

### Objectives

The study aims to gain a better understanding of clients’ experiences with the game or videos, including technical difficulties, what was helpful, what could be improved, and impact on the study variables of interest, and clinician’s impressions of the game’s impact on clients and ideas for dissemination and implementation.

## Methods

### Development of OTG

In the pilot study, we created a prototype of OTG, a game based in a fictional town where a player with psychosis has their own apartment and can go to school, work, mental health clinic, park, movie theater, and gym [12]. OTG included quests to practice real-world skills and opportunities to collect coins, information about FEP, and videos with stories of hope and recovery (ie, RVs that are in the comparison condition). Our team used participant feedback from the pilot study and the Youth Advisory Board to refine and augment the game. We held 3 web-based meetings with the Youth Advisory Board. For some members, technology was a challenge; thus, our team also provided the opportunity for members to offer written feedback about the game. On the basis of the feedback, we improved the game’s functionality (changing to a new platform that allows mobile use and developing a smartphone interface), included more quests and interactions with nonplayer characters, expanded the video library, added more customization features to the main character (hair, facial features, and body type), and provided rewards for collecting points such as opportunities to upgrade the avatar’s outfit and decorate their apartment. We also included an in-game computer with links to information about psychosis, treatment options, and wellness strategies.

As modifications were made, the Center for Social Innovation development team used Agile methodologies, working in 2-week sprints. Partners at OnTrackNY were included in weekly planning meetings to enhance and facilitate communication and stakeholder buy-in. This was critical to the development of the final product. Several times, modifications recommended by the Youth Advisory Board or that emerged from the pilot data were overruled by OnTrackNY partners to avoid content that could be traumatizing or viewed as offering *treatment* within the game. For example, in a scenario where the player had to decide what to do if a barista gives him the wrong drink at a coffee shop, we decided not to include topics such as having the player feel overwhelmed by noise and crowding in the coffee shop. At times, this created a shift away from situations in the game that were viewed as more realistic by reviewers; however, our team deferred to clinically trained staff at OnTrackNY to make this determination. OnTrackNY staff relied on evidence-based approaches to create materials and dialogues for the game. For example, a stress management handout included deep breathing, progressive muscle relaxation, and visualization. In dialogues with other nonplayer characters, social skills training principles were used, including how to have a casual conversation, how to ask for something, and how to assertively express needs. Other recommendations, such as the Youth Advisory Board’s observation that OTG would be enhanced by playing with other people, were beyond the scope and budget of this project.

Quests provided opportunities to engage in decision-making and view potential consequences of these decisions. For example, in one scenario, the player was given the wrong drink at a coffee shop and had to decide how to respond, and in another, the player had to decide whether to engage in a social interaction with a neighbor. These scenarios provided individuals with the opportunity to practice social skills, see the outcomes of potential responses, and receive immediate feedback in a safe environment mimicking potential real-world situations, designed to promote empowerment, hope, and recovery. Resources available on the in-game computer and the RVs triggered throughout the game (and comprising the RV condition) provided opportunities for psychoeducation and promoted stories of hope and recovery in individuals with psychosis. All these additions were aimed at addressing stigma, engagement, hope, recovery, and knowledge about psychosis.

### RV Condition

The RVs, present in both comparison conditions, comprised 24 videos, each with duration of 3-5 minutes, featuring individuals who received treatment in OnTrackNY and their relatives. These individuals shared their experiences living with psychosis and the challenges and successes they experienced during the recovery process. For example, in a video, a young man with psychosis and his mother described his symptoms during illness onset, the benefits of connecting with treatment, his current participation in work and school, and positive responses from others when he shared his experience.

These videos are freely available to patients and families on the New York State Psychiatric Institute Center of Practice Innovations website; however, they are not formally part of treatment as usual. A shortened 90-second version of the videos has been tested in previous RCTs and has shown reduced public stigma in an MTurk general population sample immediately after the intervention [13] and at the 30-day follow-up [14]. In the tested video, a young woman with schizophrenia described her symptoms during illness onset; her current daily difficulties, including experiencing attenuated psychotic symptoms; and her ability to participate in work and maintain meaningful relationships. Similar to other contact-based stigma reduction interventions, which provide opportunities to engage in live or video-based contact with a presenter with mental illness, the video provided participants with the opportunity to engage in video-based social contact with an individual who exhibited some attenuated symptoms of psychosis and was able to function well in her social and occupational roles, thereby, potentially disconfirming preconceived notions about individuals with psychosis and reducing stigma [15].

### Recruitment

Recruitment for the quantitative study of 159 individuals with FEP (ie, clients) occurred between April 18, 2019, and December 30, 2020. Participants completed a baseline assessment and follow-up assessments at 2 months and 5 months after enrollment. Participants were initially recruited from 18 clinical sites in the OnTrackNY network, a CSC program in New York State. Owing to recruitment challenges imposed by the COVID-19 pandemic, 35.8% (57/159) of participants were recruited via the web from various CSC programs across the country. Inclusion criteria were the following: a diagnosis of nonaffective psychosis and receiving services at a CSC program, aged 16-30 years, can speak English, able to give fully informed consent, access to the internet with a PC or tablet (sites provide access to computers or tablets on-site if this is a barrier), and access to email. Participants provided consent, which included a question that asked whether they would give permission to be contacted to participate in a qualitative interview at a later time.

Study participants were randomly assigned to the OTG or RV intervention. Participants were provided basic instructions on how to access and navigate the intervention to which they were assigned and were given 2 months to play the entire game or watch all RVs. They were also encouraged to use OTG or RV on a weekly basis throughout that 2-month period. Our research team collected analytic data on the extent to which each participant accessed OTG or the RV site and the content within each site. Data reflect cumulative use and indicate that approximately 30.2% (48/159) of the participants accessed the sites they were assigned. Inconsistent use, possible reasons for lack of use, and overall implications will be discussed in a subsequent manuscript on outcomes related to the study. For qualitative interviews, the team used purposive sampling, examining use data to identify individuals with a range of exposure to the intervention. Interviewing participants who varied in product use allowed the team to identify barriers to and facilitators of use.

For the qualitative study, a subsample of participants from the OTG and RV groups who agreed to be contacted were recruited between July 22, 2020, and December 1, 2020. We planned to enroll 20 clients in 1:1 ratio (eg, 10/20, 50% OTG participants and 10/20, 50% RV participants). Of the 42 individuals who provided consent to be contacted for the qualitative study, 93% (39/42) were contacted, and 41% (16/39) of them agreed to participate in the interview (5/16, 31% in the RV group and 11/16, 69% in the OTG group). Of the 23 participants who were contacted but did not participate, 13% (3/23) had difficulties with scheduling, 35% (8/23) declined, and 52% (12/23) did not respond or had invalid contact information. Unfortunately, we faced difficulties in engaging participants from the RV group. Some RV participants indicated they did not want to stay involved in the study after finding out they would not be able to play OTG and some participants did not want to complete additional assessments.

A total of 9 clinicians from CSC programs (n=1, 11% with a client in RV group and n=8, 89% with clients in both OTG and RV groups) were enrolled in the qualitative study between June 25, 2020, and August 31, 2020. Some clinicians involved in the study chose not to participate in the qualitative interviews. Inclusion criteria were being a licensed mental professional providing care in a New York CSC program and working with a client participating in the quantitative study.

### Ethics Approval

All procedures were approved by the New York State Psychiatric Institute Review Board (protocol #7643) and the trial was registered on Clinicaltrials.gov ID # NCT03390491.

### Qualitative Interviews

Interviews were conducted by 2 trained research assistants (Terriann Nicholson and Sapna Mendon-Plasek) via WebEx (Cisco) and were audio-recorded. Interviews lasted approximately 30 minutes, and OnTrackNY participants were compensated US $25. Clinicians were not compensated. Brief semistructured interview guides were used for participants and clinicians. Client interview questions were intended to elaborate on quantitative measures and included questions about the game’s influence on treatment engagement; recovery; empowerment; hope; understanding of psychosis; and stigma, which was divided into self-stigma and public stigma. Self-stigma encompasses the judgments and negative beliefs people internalize or hold about themselves [16]. Public stigma encompasses the attitudes and feelings expressed by many people in the public toward individuals with psychosis [16]. It involves identifying differences, connecting those differences to stereotypes, and separating groups by those stereotyped differences. Questions corresponding to each domain are included in Textbox 1. Additional questions were related to potential technical difficulties, most favorite and least favorite aspects, frequency of use, relevance to experiences with symptoms and treatment, and recommendations for improvement.

Clinician interviews included questions about the following: whether the game or videos were discussed in the session, most helpful or least helpful aspects, perceived changes in client interactions with the team and their attitudes, whether they would recommend the game or videos to other clients, perceived barriers to client use, ways to encourage individuals to use the products, and recommendations for improvement.

**Client qualitative interview questions and domains assessed.**

**Engagement**

- Thinking back to before you started playing the game or using the recovery video site and after, has your participation in treatment and other support services changed?

**Recovery**

- Let’s talk about your feelings about recovery before playing the game or using the videos website and after. When you think back, have there been any changes in how you think or feel about recovery or do you feel the same?

**Empowerment**

- Did playing the game or using the videos site help you think about ways to speak up for yourself?
- Did you think more about how you can participate in and make decisions about your treatment?

**Hope**

- Let’s talk about your hope for the future and your treatment before playing the game or using the videos site and after. When you think back, have you felt any changes in your hopefulness for the future or do you feel the same?

**Understanding of psychosis**

- How was the content relevant to your own life and experiences with your symptoms and treatment?
- Did you learn anything about your symptoms or treatment?

**Self-stigma**

- After playing the game or using the videos site, did you feel differently about yourself?

**Public stigma**

- How do you think the game or videos site portrayed individuals living with mental illness?

### Data Analysis

For qualitative data, the rapid identification of themes from audio recordings method was used [17]. Similar to procedures in our pilot study [12], a summary template was created by one of the authors (FM) with the predetermined interview codes described above. Codes for the client data included the following: treatment engagement, recovery, stigma, empowerment, hope, understanding of psychosis, how often game or videos were used, likes and dislikes, and technical difficulties. Codes for clinician data included the following: most and least helpful aspects of game or videos, changes observed in the client, barriers and ways to improve engagement, whether they would recommend the game or videos, and additional feedback. Upon completion of interviews, the authors listened to the recordings and documented key messages and relevant quotes. Then, the data were categorized into common themes across study conditions and differences across study conditions and respondent roles. Some themes reflected *a priori* topics from the template and others emerged directly from the interviews. Another author (ED) revised the proposed codes, added new themes, and synthesized the results. Both coders used a focused coding approach to determine which topics arose often and which represented unusual or particular concerns. Disagreements between coders were resolved by discussion among all authors.

## Results

### Sample Characteristics

On the basis of self-reported data from participants in the qualitative subsample, the clients’ mean age was 21.81 years, and 56% (9/16) of the them were men. Participants identified as Black or African American (6/16, 38%), White (5/16, 31%), and Asian (1/16, 6%), and approximately half of them were Hispanic (7/16, 44%). There were no significant differences between RV and OTG groups at baseline (Table 1). All participants were from New York CSC sites. Demographic data were not collected for clinicians.

**Table 1:** Demographic characteristics for recovery video and OnTrack>The Game groups.

Characteristics		Treatment group		Comparison					
		OnTrack>The Game group (n=11)	Recovery videos group (n=5)	Chi-square test (*df*)		*t* test^a^ (*df*)		*P* value	
**Sex, n (%)**					0.04 (1)		N/A^b^		.84
	Men	6 (55)	3 (60)						
	Women	5 (45)	2 (40)						
Age (years), mean (SD)		21.55 (4.01)	22.40 (1.95)	N/A		0.57 (13.79)		.58	
**Race, n (%)**					1.9 (3)		N/A		.60
	White	3 (27)	2 (40)						
	Black or African American	5 (45)	1 (20)						
	Asian	1 (9)	0 (0)						
	Unknown	2 (18)	2 (40)						
**Ethnicity, n (%)**					0.04 (1)		N/A		.84
	Yes, Hispanic or Latino	5 (45)	2 (40)						
	Not Hispanic or Latino	6 (55)	3 (60)						

^a^A 2-tailed *t* test.

^b^N/A: not applicable.

### Similar Themes for Clients Across Study Conditions

#### Overview

The analysis generated data related to 6 main themes (Textbox 2). The first 4 themes demonstrate the benefits of client exposure to OTG and RV content. These included (1) access to depictions of relatable experiences that provide individuals with psychosis with models of possibility, (2) increased sense of hope for the future and the ability to advance through recovery, (3) decreased self-stigma and public stigma, and (4) increased understanding of the importance of family and social support and their inclusion in the treatment process. The final 2 themes demonstrate differences between user experiences in the OTG and RV conditions. Improved empowerment was identified only by OTG participants, and clinicians strongly valued use of RVs over OTG with clients.

**Key themes and subthemes identified.**

**OnTrack>The Game (OTG) and recovery videos (RVs) are relatable.**

- The vast majority of OTG or RV participants report that the content is relatable to them and other individuals with psychosis.
- OTG and RV provide accurate representation of real-life experiences and daily challenges for individuals with psychosis.
- OTG provides helpful tools for self-reflection for individuals with psychosis.

**OTG and RVs improve feelings of hope for the future.**

- The vast majority of OTG participants report that the game increases their sense of hope for their future and their ability to live, work, and advance their lives as a result of their participation.
- The majority of RV participants report that the RVs increase their sense of hope for their future and their ability to advance through recovery.

**OTG and RVs decrease self-stigma and public stigma.**

- OTG and RV participants report a slight decrease in both self-stigma and public stigma.
- Although most OTG and RV participants report a decrease in self-stigma and public stigma, there are some mixed results. For example, some OTG participants note that individuals with psychosis are portrayed “too favorably” by the game.

**A circle of support is important.**

- OTG and RV participants report an increased understanding of the importance of family and social support.
- OTG and RV participants report an increased understanding of the importance of involving the people who are part of your support system in your treatment.

**Improved empowerment is identified only by OTG participants.**

- Most OTG respondents report that the game increases their feelings of empowerment.
- This theme did not emerge for RV participants.

**Clinicians strongly value use of RVs over OTG for clients.**

- All clinician respondents would recommend the RVs to future clients.
- Slightly more than half of the clinicians would recommend OTG to future clients.

#### Relatability

Most study participants reported that both OTG and RVs were relatable. Totally, 64% (7/11) of the OTG participants and 80% (4/5) of the RV participants reported that they related to the content and felt enhanced feelings of connection and understanding after engaging with it. Participants provided many examples of the potential for this content to enhance feelings of connection and understanding. They reported feeling less lonely or isolated. OTG and RV participants also reported that OTG or RV provided accurate representation of real-life experiences and daily challenges for individuals with psychosis. Furthermore, OTG participants reported that the game provided helpful tools for self-reflection for individuals with psychosis:

[The RVs] help identify some of the struggles that come with psychosis and gives helpful tools to help with challenges, especially parts that talk about disclosure and when it’s appropriate to share.

RV participant

Seeing daily activities in the midst of anxious situations, made conventional life more relatable. Learned a lot about myself, a great tool to be reflective.

OTG participant

I feel different. I feel hopeful for the future. The goals in the game, such as getting up and taking care of yourself, made me more aware of what I was doing daily.

OTG participant

#### Improved Feelings of Hope

Most of both OTG and RV participants (15/16, 94%) reported increased feelings of hope. All the OTG participants (11/11, 100%) noted an increased sense of hope for their future and increased their ability to live, work, and advance their lives as a result of their participation. OTG aided participants in addressing some of their self-doubt and beginning the process of overcoming it. Of the 5 RV participants, 4 (80%) participants reported that the RVs increased their sense of hope for the future and their ability to advance through recovery:

They had a diagnosis and still continued their life; at the beginning, I thought ‘that’s it, I’m not going to live a normal life’; now I feel proactive and am in school; looking at participants in the game helped.

OTG participant

They’ve been through episodes, but overcame them, continue living their life -- that made me feel like I can live a life.

OTG participant

I am more hopeful after watching the videos, and pretty much whenever I have connection with participants with mental illness. It made me realize that there is always another side no matter what difficulty you are going through...I feel more hopeful when I see success stories and I see the artwork they create. It reinforced the things I knew before. Even if you are in hell there is light at the end of the tunnel.

RV participant

#### Decreased Self-Stigma and Public Stigma

Most OTG and RV participants reported increased positivity and a decrease in both self-stigma and public stigma as a result of playing OTG and watching the RVs. In all, 73% (8/11) of the OTG participants and 100% (5/5) of the RV participants noted that exposure to content that portrays individuals with psychosis positively, as complex people with talents and goals, not only helped them to cope with their own diagnosis but also to judge others with mental illness less harshly:

It gave me positivity that mental illness is nothing bad. Whatever happened, I went through it and survived.

OTG participant

[Individuals with psychosis] were portrayed very positively and honest and genuine. They spoke very openly about their experiences and how they overcame it. When I finished all the videos, I reflected on what I saw and heard on the videos and it was very inspiring. I felt very happy and positive about it, seeing people in my age group going through the same struggles and overcoming it.

RV participant

Although most OTG and RV participants reported a decrease in self-stigma and public stigma, some OTG participants reported that the game portrayed individuals with psychosis “too favorably.” They suggested that OTG include content where characters are struggling or not feeling well, which would allow those players to see examples of how those characters practice self-care and help themselves come out of difficult situations:

The game didn’t really show what happens in my own mind. It didn’t portray the individual’s feelings and thoughts outside of communicating with others. It was portrayed too positively. Nothing was negative or showed obstacles.

OTG participant

#### The Importance of a Circle of Support

Many OTG and RV participants reported an increased understanding of the importance of family and social support for individuals with psychosis. Furthermore, participants reported an increased understanding of the importance of involving the people who are part of your support system in your treatment. Both the OTG and RVs emphasize the pivotal importance of relationships in healing and recovery. The content encourages users to identify family, friends, partners, groups (eg, support groups, study groups, and sports teams), mental health and healing practitioners (eg, primary clinician, peer specialist, and acupuncturist), mentors (eg, leader of a youth group, work supervisor, friend of the family, and teacher or professor), support animals, and any other outlets that are supportive. RVs and an activity sheet helped users consider the people who are part of their circle of support and develop effective ways to strengthen those relationships:

[The RVs] made me realize the importance of a support system. It affected my treatment because prior, when I had symptoms I isolated [my]self and psychosis is hard to understand. So, a support system was important because it helped bring back reality.

RV participant

### Differences Across Study Conditions

Although several similar themes were identified across OTG and RVs, important differences were also evident. Most notable were participants’ perspectives on the impact of the interventions on empowerment. The theme of empowerment did not emerge for RV participants. Of the 11 OTG participants, 10 (91%) participants noted that OTG increased their feelings of empowerment in treatment by showing examples of how to speak up during treatment and elsewhere and by presenting models of possibility in the form of these characters who are dealing with some of the same challenges as our participants and still building rich, fulfilling lives:

Even before I was involved with treatment, the game showed me I have to be involved—helped me become involved in decisions that doctors were making about me.

OTG participant

I actually have a say in my treatment; the game helped me realize that I have to advocate for myself and have to be honest, I can control what happens in my treatment.

OTG participant

### Differences in Clinician and Client Perspectives

The most notable difference between clinician and client perspectives of the interventions was related to the value of RVs and OTG. *Clinicians overwhelmingly stated that they would recommend RVs over OTG to clients; however, client preferences were mixed***.** All the clinicians interviewed during the study (9/9, 100%) noted that they would recommend the RVs to future clients, but only 56% (5/9) of them stated that they would recommend OTG. They cited issues such as difficulty for clients to access adequate computers and internet and computer skills of clients as barriers to use. They also noted that OTG graphics and technology had become outdated and the graphics were not well-suited for the key audience: “Some clients were uncomfortable with OTG’s graphics and felt they were intended for a younger audience” [clinician participant].

Clients also reported some challenges with gameplay. Some clients had difficulty in understanding how to move from one level to another:

I would complete a task and when I went back it would be erased and I would have to repeat a task that was already completed.

OTG participant

Sometimes choices weren’t clear, so participants had to choose every option just to advance.

OTG participant

Although clinicians found more value in the RVs, results were mixed at the client level, and some participants shared that they found it difficult to find time to watch the RVs and that the videos themselves were “too long.” Furthermore, the interviewed clients had a more positive view of OTG than the interviewed clinicians. Clients expressed enthusiasm about many different facets of OTG: being able to create or personalize their own character, the game’s portrayal of the decision-making process and the ability to make decisions within the game, and the game’s messaging around communication skills and how to manage daily-life commitments.

### Additional Implementation Considerations

#### Overview

In addition to themes described above, important considerations should be made for adoption and implementation of OTG and RVs in the field. The research team asked clinicians to provide suggestions regarding how to improve client engagement with the products tested in this study. The suggestions described below will steer future implementation and help our team maximize resources and achieve wider reach with OTG and RVs.

In their suggestions, clinicians were considering two questions: (1) How can OTG and RVs be made more accessible to clients? and (2) How can OTG and the RVs be integrated into clients’ overall treatment plans?

#### Accessibility

To make OTG and RVs more accessible, clinicians suggested providing access to computers or tablets for clients, hosting a demonstration of OTG and RVs on the OnTrackNY website, having on-site peers disseminate OTG and RVs while providing access to computers or tablets, and making OTG and RV resources available to families of clients using the technology so they can encourage them to use the products. Clinicians also had recommendations for making OTG and RVs easier to find. For example, they suggested setting up an Instagram page for OTG and RVs that clients could follow or, perhaps, advertising with videos that feature individuals who have played the game to build some familiarity. In addition to suggesting the creation of social media accounts for OTG and RVs, clinicians also suggested having OnTrackNY advertise OTG and RVs more in their social media campaigns.

#### Integration With Services

Clinicians provided suggestions for seamless integration of OTG and RVs into the clients’ treatment. They recommended using OTG and RVs as training tools when onboarding providers; enabling providers to use OTG and RV, so that they can help orient clients to the technology and discuss how it fits into their treatment; playing the game or watching the RVs during home visits; and providing examples of peer feedback from other participants who have used OTG and RV, so that it feels more recognizable. Clinicians also suggested using OTG and RVs to facilitate communication during early sessions, when rapport is still being built: “Sometimes it is hard for participants to talk and it would be nice to be able to play the game” [clinician participant].

## Discussion

### Principal Findings

This study qualitatively examined the impact of OTG and RVs on engagement, stigma, empowerment, hope, recovery, and understanding of psychosis in clients receiving CSC. Common themes for clients included relatability, increased hope and possibility of recovery, decreased self-stigma and public stigma, and increased understanding of the importance of family and social support, and OTG participants reported increased empowerment. Clinicians strongly preferred RVs and offered suggestions for dissemination and implementation for both OTG and RVs, including providing clients with computers or tablets, advertising the products through social media or a website, having families facilitate dissemination, and using the products during onboarding of providers and during initial sessions with clients.

Relatability of content was an expected finding as a Youth Advisory Board was involved in game development and refinement, and feedback was incorporated from pilot participants. The newest iteration of the game included resources about FEP and more family and friends characters and provided the option for characters to make poor decisions and experience the consequences of those decisions [12]. A notable and interesting finding included that some OTG participants believed that the game portrayed individuals with psychosis “too favorably.” This finding suggests that participants would like to see a balance of positive and negative experiences that individuals face.

The positive impact the games or videos had on improving hope and reducing both self-stigma and public stigma are also notable. These findings are consistent with previous research, which found that the RVs led to reduced public stigma in the general population [13,14]. Previous gaming research also suggests that video game avatars have the potential to reduce stigma through transportation into the story line and identification with the avatar [10]. It is possible that exposure to content that portrayed individuals with psychosis in a positive light and allowed participants to take on the *identity* of this character equipped them with the ability to cope with their own diagnosis and be less stigmatizing toward others with mental illness. This may have also contributed to the increased sense of hope noted by participants.

A finding that emerged outside our predetermined themes included an increased understanding of the importance of social support and their involvement in treatment. Previous research suggests that social support is particularly important during FEP and can correlate with low levels of positive symptoms and few hospitalizations [18]. In addition, a large number of individuals with psychosis have poor perceived support and are susceptible to feelings of loneliness and anxiety [19]. The game or videos may encourage participants, particularly those who tend to withdraw when they are symptomatic, to stay connected with individuals in their social network and involve them in treatment.

It was interesting to note that the theme of empowerment emerged in OTG but not in the RV group. Although this may have been owing to insufficient sample size in the RV group, it is also possible that specific game elements, such as providing knowledge, supporting behavior, and providing skills training, contributed to increased empowerment [20]. The game provided knowledge about psychosis and provided individuals the opportunity to learn from stories of other individuals with psychosis through the RVs. It also taught participants skills applicable to daily life, such as assertiveness, and supported behavior by providing players with choice in dialogues with nonplayer characters and direct feedback on these choices. Although the RV group also provided knowledge and skills training in the form of handouts and the videos, the supportive behavior component was lacking, as participants did not have the opportunity to engage in a choice and immediately see consequences or receive corrective feedback.

Notable findings also emerged regarding dissemination and implementation; clinicians had a preference for RV over OTG, but clients did not. Clinicians cited access issues, lack of computer skills, outdated and immature graphics, and lack of understanding of how to move from one level to another as common reasons. It was surprising that interviewed clients had a more positive view of the game. Clinicians may have overestimated how much these factors were impacting participants or the participants experiencing difficulties may not have participated in interviews. In the future, as clinicians may be the ones introducing OTG and RV to clients, it is important that they feel the materials are relevant and accessible to participants.

Importantly, many of the issues that were described reflect challenges identified by the Nonadoption, Abandonment, Scale-up, Spread, and Sustainability framework, which aims to help researchers predict and identify challenges in the implementation of technology in health care [21]. Factors to consider when implementing new technologies include the complexity of the health condition being treated, consumers’ sociocultural aspects that influence product use (eg, lack of access to computers), ease of use of the product and esthetics (eg, clunkiness), perceived value of the product, whether staff and consumers are willing or able to adopt the product, organizational capacity and readiness, work involved in product implementation, and wider institutional and sociocultural context influencing spread and sustainability [21]. To address concerns in our study regarding access and use, clinicians suggested providing participants with technological devices; having demonstration versions or having a peer, family, or clinician aid in dissemination; and using social media to advertise the products. Suggestions to have peers or clinicians aid in dissemination are consistent with calls for digital navigators to aid in the integration of technology into health care [22]. Although providing clients with technological devices may not be the most cost-effective solution for all clinics, these resources are available in OnTrackNY clinics. Clinicians also suggested that OTG and RV could be used to facilitate rapport-building and communication during initial treatment engagement. Perhaps, clients and clinicians could watch the videos or play the game together or they could be assigned as between-session homework and various topics could be discussed during the following session.

### Limitations

This study had some limitations. The small sample of clients and clinicians may limit the generalizability and reliability of study findings. The small number of individuals in the RV group made it difficult to determine whether the difference in empowerment observed in the OTG was owing to the intervention or whether this theme would have emerged in the RV group also. Another potential limitation is that as all the participants were enrolled in OnTrackNY and were in different stages of treatment, it was unclear whether findings that emerged were owing to the interventions or being part of a treatment program. In addition, both groups had access to the same RVs (with RVs embedded within OTG); thus, it is difficult to distinguish differences that might have emerged from both groups.

### Conclusions

It is important to note that despite these limitations, clear themes emerged that can help generate hypotheses and future research in this area. This study was able to highlight similar and differing themes in the experiences of clients and clinicians in using OTG and RV. Future research should aim to explore the dissemination and implementation of OTG and RV and the impact of these interventions on participants who are not enrolled in FEP programs. Other studies should also explore the differential impact of the intervention on clients at various stages of treatment and the impact of these products on family members or individuals without psychosis. The findings of our quantitative study will be reported separately.

## Appendix

Multimedia Appendix 1 CONSORT-eHEALTH checklist (version 1.6.1).

## Abbreviations

CSC: coordinated specialty care
FEP: first episode psychosis
OTG: OnTrack>The Game
RCT: randomized controlled trial
RV: recovery video
